# Understanding the patient and supporter journey in cocaine use disorder

**DOI:** 10.3389/fpsyt.2024.1230626

**Published:** 2024-04-10

**Authors:** Denise Leclair, Katherine M. Waye, Baltazar Gomez-Mancilla, Brian D. Kiluk, Ananda Krishna Karanam, Partha S. Banerjee, Velusamy Shanmuganathan Muthusamy, Suzanne Maahs

**Affiliations:** ^1^Novartis Pharmaceuticals Corporation, One Health Plaza, East Hanover, NJ, United States; ^2^Novartis BioMedical Research, Cambridge, MA, United States; ^3^Novartis BioMedical Research, Novartis Pharma AG, Basel, Switzerland; ^4^McGill University, Montreal, QC, Canada; ^5^Yale School of Medicine, New Haven, CT, United States; ^6^Novartis Healthcare Pvt. Ltd., Hyderabad, India

**Keywords:** cocaine, cocaine use disorder, journey, online bulletin board, patient perspectives, qualitative research, supporters

## Abstract

**Background:**

There is a paucity of literature describing experiences and journey of individuals with cocaine use disorder (CUD) and supporters who care for them. The aim of this study was to understand and document the journey of individuals with current CUD, those in CUD remission, and supporters.

**Methods:**

The online bulletin board (OBB) is a qualitative tool where participants engage in an interactive discussion on a virtual forum. After completing a 15-minute screening questionnaire determining eligibility, individuals in CUD remission and supporters participated in an OBB for 60 minutes, split across 8 days over 2 weeks. Individuals with current CUD participated in a one-time virtual focus group discussion for 90 minutes.

**Results:**

Individuals in CUD remission (n=35) were from Brazil, France, Spain, the UK, and the US; those with current CUD (n=5) and supporters (n=6) were from the US. Key insights were that individuals with current CUD were seeking a ‘euphoric high’ that cocaine provides. Those in CUD remission described a ‘euphoric high’ when they first tried cocaine, but over time it became harder to re-create this feeling. Individuals in CUD remission expressed a ‘rollercoaster’ of emotions from when they first started using cocaine to when they stopped. Supporters were sad, isolated, and worried about a potential cocaine overdose for their loved ones with CUD.

**Conclusion:**

The study provides valuable insights into the experiences and journey of individuals with CUD and their supporters. Data generated from this study gives insights into this under-served and growing population.

## 1 Introduction

Cocaine use disorder (CUD) continues to be a serious global health problem. Globally, an estimated 20 million people used cocaine in 2019, which corresponds to 0.4% of the world’s adult population aged 15–64 years (1). Currently, there is no approved pharmacotherapy for CUD (2, 3). Future treatments should consider the unique perspectives and preferences of patients as well as their supporters to help inform drug development and regulatory decision making (4).

Patients have always been the reason for drug development, however, their perspectives were not always included. Recently, the US Food and Drug Administration (FDA) has increased efforts to integrate the patient voice into the drug development process through Patient-Focused Drug Development initiative (4). However, there remains a critical unmet need to understand the patient journey as a way to guide drug development.

The perspectives of supporters are equally important as those of patients (4), and are poorly understood and underrepresented in the literature. Supporters can play a central role in the effective recovery of individuals with substance use disorder (SUD) and they experience a high emotional burden (5); but little is known about the supporters’ perspectives in CUD. In this study we have, therefore, recruited supporters in line with recommendations from the Food and Drug Administration (FDA) guidance (4) as their perspectives are critical to treatment support and recovery for those with CUD.

Researchers can now engage individuals with CUD and their supporters virtually using an online bulletin board (OBB) qualitative approach. OBBs represent asynchronous qualitative market research tools that provide an open, private forum for interactive discussion among participants (6). Several studies have been conducted using the OBB method to provide insights into different health conditions (6–10), but this approach has not been previously used in CUD. In the present study, we used the OBB qualitative approach to gain understanding and document the experiences of individuals with current CUD, those in CUD remission, and supporters.

To date, there is a gap in the knowledge regarding the personal experiences and the journey of patients with CUD and the supporters who care for them. The aim of this study was therefore to understand and document the journey of individuals with current CUD, those in CUD remission, and their supporters. Insights obtained in the OBB to help inform clinical trial approaches for CUD are, however, the subject of another publication article.

## 2 Methods

### 2.1 Study design and setting

The study was conducted over 2 weeks between February and March 2021 across Brazil, France, Spain, the UK, and the US. This study included 2 qualitative approaches: (i) 5 OBBs with individuals in CUD remission and 1 OBB with friends or family members (supporters) of someone with CUD as well as (ii) a 90 minute structured focus group with individuals living with CUD.

In this study, a mix of recruitment methods were used to enroll the target population, including contacting participants on market research panels, social media advertising in relevant special-interest groups, and referrals from volunteers at rehabilitation clinics.

A detailed description of the analyses and study methods is published elsewhere (11) (See **Appendix 1** for study procedures and **Appendix 2** for anonymity of data. Briefly, a screening questionnaire (for 15 minutes) assessed eligibility for participation. After completion of the screening questionnaire (**Appendix 3**), individuals in CUD remission and supporters participated in a structured OBB for a total of 60 minutes hosted by a trained moderator in the native language. The OBB was split across 8 days over 2 weeks. Individuals with current CUD participated in a structured virtual focus group discussion for 90 minutes in a single day hosted by a trained moderator. Participants received a modest compensation for their time and contribution to the study.

### 2.2 Cocaine use disorder: OBB and focus groups

The study included the following groups (i) one focus group in the US with participants diagnosed with CUD (individuals with current CUD); (ii) five OBBs were conducted in Brazil, France, Spain, the UK, and the US with participants who had recovered from CUD (individuals in CUD remission); and (iii) one OBB in the US with close friends or family members of someone who used cocaine regularly (supporters).

### 2.3 Study participants

Participants answered the screening questionnaire (15 minutes) to ensure their eligibility and willingness to participate in the study (see **Appendix 3** for screening questionnaire). During the screening period, to qualify as individuals with current CUD, participants had to report the use of ‘cocaine’ or ‘crack cocaine’ regularly, or report a diagnosis consistent with CUD or SUD, or must have answered ‘yes’ to at least five Diagnostic and Statistical Manual of Mental Disorders, Fifth Edition (DSM-5) criteria questions about CUD to ensure individuals with at least moderate CUD were included. Individuals in CUD remission must have answered ‘yes’ to regular use of ‘cocaine’ or ‘crack cocaine’ in the past or had to report a history of substance use including ‘cocaine’ or must have answered ‘yes’ to at least five DSM-5 criteria questions to ensure inclusion of individuals with at least moderate CUD. Supporters were eligible to participate if they had indicated they were caring for or supporting someone currently using ‘crack cocaine’ or ‘cocaine’. They must have answered ‘yes’ to at least five DSM-5 criteria questions about their loved ones or confirmed a history of diagnosis consistent with CUD or SUD.

### 2.4 Conduct of the study

The study was conducted in partnership with an external vendor specializing in market research methodologies. The study was conducted in accordance with the Declaration of Helsinki and the guidelines for Good Epidemiology Practice. Standard procedures were adhered to for the protection of participants’ rights, including written informed consent, data privacy, anonymity, and the right to withdrawal, as well as thorough assessment of qualification of the vendor was undertaken.

### 2.5 Ethical approval

Institutional Review Board approval was not sought because this was a market research study. Written consent was obtained from individuals before study participation. Participants had the right to withdraw at any time during the study.

### 2.6 Qualitative analysis of participant responses

The main output for the OBB and the virtual focus group was a transcript of the discussion. The OBB also generated some additional outputs, such as images and responses to closed questions (eg., agreement with statements or polling) which were analyzed alongside the transcripts. The anonymized data underwent thematic analysis. Transcripts were reviewed iteratively by several team members to identify and examine key themes and patterns within interviews, within and across markets and patient demographics.

## 3 Results

### 3.1 Individuals with current CUD

**Figure 1:**
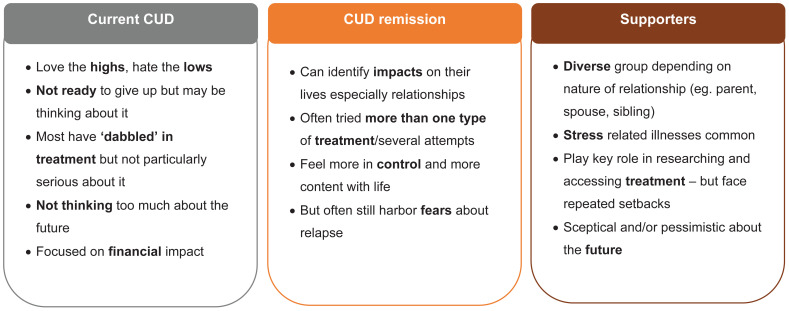
Feedback about the impact of using cocaine in individuals varied between groups (current cocaine use disorder [CUD], those in CUD remission, and supporters).

Individuals with current CUD had a diagnosis consistent with CUD or SUD during screening. They were men (n=5) aged 22 to 49 years participating from the US (**Table 1a**). They described an attachment and a feeling of a ‘euphoric high’ that cocaine provides. These individuals were most interested in something ‘new’ and were focused on liking the ‘joyful feeling’ of escape from using cocaine. They did not feel ready to give up cocaine, and most of them felt the impacts of CUD as relatively minor or reversible especially compared to other drugs (**Figure 1**).

Table 1Demographics of individuals with **(a)** current cocaine use disorder (CUD), **(b)** those in CUD remission, and **(c)** supporters.

**Table:** 

(a)	
	The US
**Gender**	5 men
**Age, years**	22–49
**Frequency of cocaine use**	More than once a day Daily At least once a week
**Treatments tried**	• Counselling/psychological support • In-patient detox or rehabilitation • Cognitive-behavioural therapy

**Table:** 

(b)					
	The US	The UK	Spain	France	Brazil
**Gender**	9 Women 3 Men	4 Women 2 Men	1 Women 5 Men	2 Women 2 Men	2 Women 5 Men
**Age, years**	22–65	30–49	30–60	23–50	30–60
**Time in recovery (range)**	35 days – 10 years	1 month – 14 years	3 years – 18 years	2 months – 10 years	1 year – 24 years
**Example occupation**	• Peer recovery coach • Drug and alcohol counsellor • Community health worker	• Student nurse • Commercial property acquisitions • Employee at a mental health charity	• Actor and model • Hospitality industry • Pensioner	• Social psychology student • Teacher • Department of health employee	• Children’s special needs teacher • Athlete • Biologist- organic food producer

**Table:** 

(c)	
	The US
**Gender**	6 Women
**Age, years**	33–53
**Relationship with CUD subject**	Aunt, cousin, sister, friend, spouse, parent
**Living with CUD subjects**	5 supporters live with CUD subjects

Cocaine use was driven by a range of factors: desire for confidence, impulsivity, coping with peer pressure, combating low mood/depression, and youthful naivety. Of the factors listed, a desire for confidence and impulsivity were the most identified drivers contributing to cocaine use. A participant shared that they were willing to try anything when first using cocaine, “I was open to anything, I had done cigarettes and pot and alcohol, anything you put in front of me, I’d was like, ‘Oh yeah let’s do that’.” Participants also reported associating cocaine with other potentially negative effects like: money spent on the drug, low mood and a ‘crash’ following use, impact on relationships with friends and family members, feeling isolated from normal life and those not using drugs, and feelings of anxiety and paranoia. They discussed the perceived negative impacts of money spent on the drug, low mood and a ‘crash’ following use, and impact on relationships with friends and family members most frequently. For example, focus group participants shared, “…you’re all really tired, sleepy the next day … so that makes me think, why am I doing this?” as well as, “it’s made me more closed off to people who don’t know or who don’t do it, I kind of would shy away.”

Individuals with current CUD experienced negative impacts of cocaine use on their health (both mental and physical), mostly centered around anxiety and damage to their nose. The negative effects on their mental health included anxiety and paranoia that worsened with cocaine use. They disliked the ‘crash’ i.e., feeling depressed and drained following cocaine use. The negative effects of cocaine use on their physical health included concerns about damaging their nose with possible signs of nose bleeds, a deviated septum, and nasal congestion from cocaine use. Once these individuals started to ‘come down’ from the high while using cocaine they also reported beginning to experience some uncomfortable withdrawal symptoms, including shaking.

### 3.2 Individuals in CUD remission

**Figure 2:**
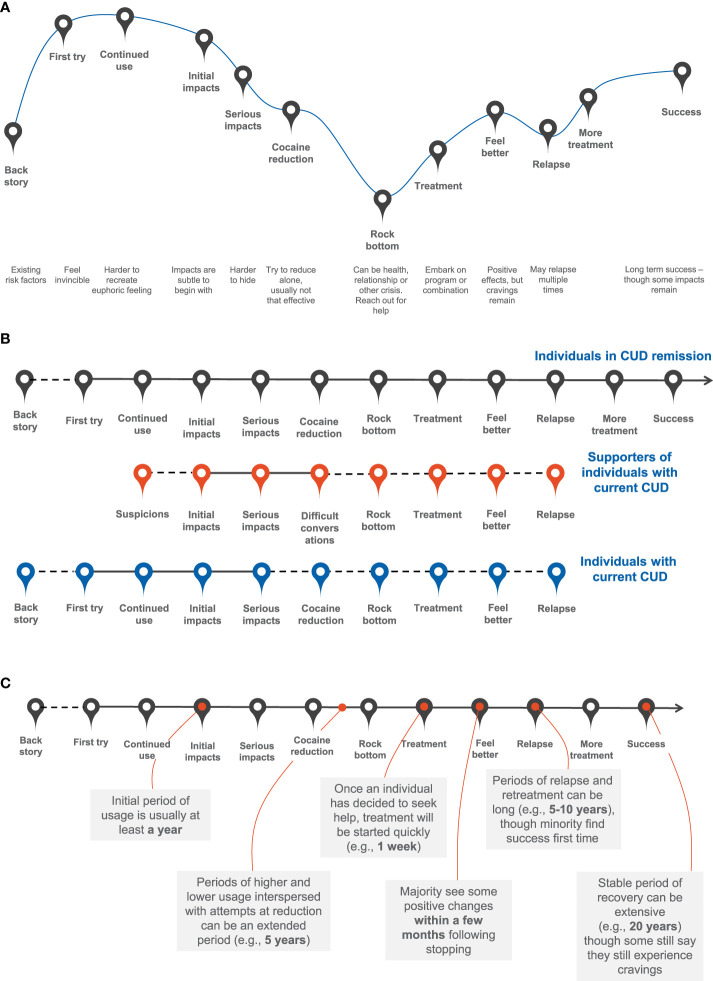
Cocaine use disorder (CUD) remission: **(A)** ‘story arc’ of participants’ journey until they reach success, **(B)** journey of supporters and individuals with current CUD and **(C)** timescale.

Among the OBB participants, 94% of those in CUD remission had a diagnosis consistent with past CUD or SUD during screening. The remaining 6% of those in CUD remission not meeting diagnostic criteria for past CUD or SUD reported regular ‘crack cocaine’ or ‘cocaine’ use in the past. The average time to diagnosis ranged 4 to 25 years. Participants ranged from being in either partial or full remission, and reported anywhere from about 1 month to 24 years of remission (**Table 1b**). Individuals in CUD remission were men (n=17) and women (n=18) (aged 22 to 65 years) from Brazil (n=7), France (n=4), Spain (n=6), the UK (n=6), and the US (n=12) (**Table 1b**). They expressed a ‘rollercoaster’ of emotions from when they first started using cocaine to when they stopped (**Figure 2**). For example, participants shared that when they first tried cocaine they had generally positive emotions like feeling impressed, free, great, alive, exhilarated, part of the group, euphoric, and anxious. However, as cocaine became part of their life and it was hard for them to step back from regular cocaine use, participants shared feeling depressed, paranoid, and powerless. Now that the participants are in recovery, they share the feeling that their life is more calm, better, and more tranquil.

The sense of feeling lost or frightened without access to cocaine was a common theme. The participants reported feeling a need for cocaine to function and that there was no life without cocaine. A key driver for those who first tried cocaine was the need to belong to a group, but later the fear of not fitting in and not being accepted in the group if they were to stop using cocaine drove continuous use. Furthermore, cocaine use was further ingrained in their lives when participants used other illicit drugs such as heroin and alcohol in conjunction with cocaine.

**Figure 3:**
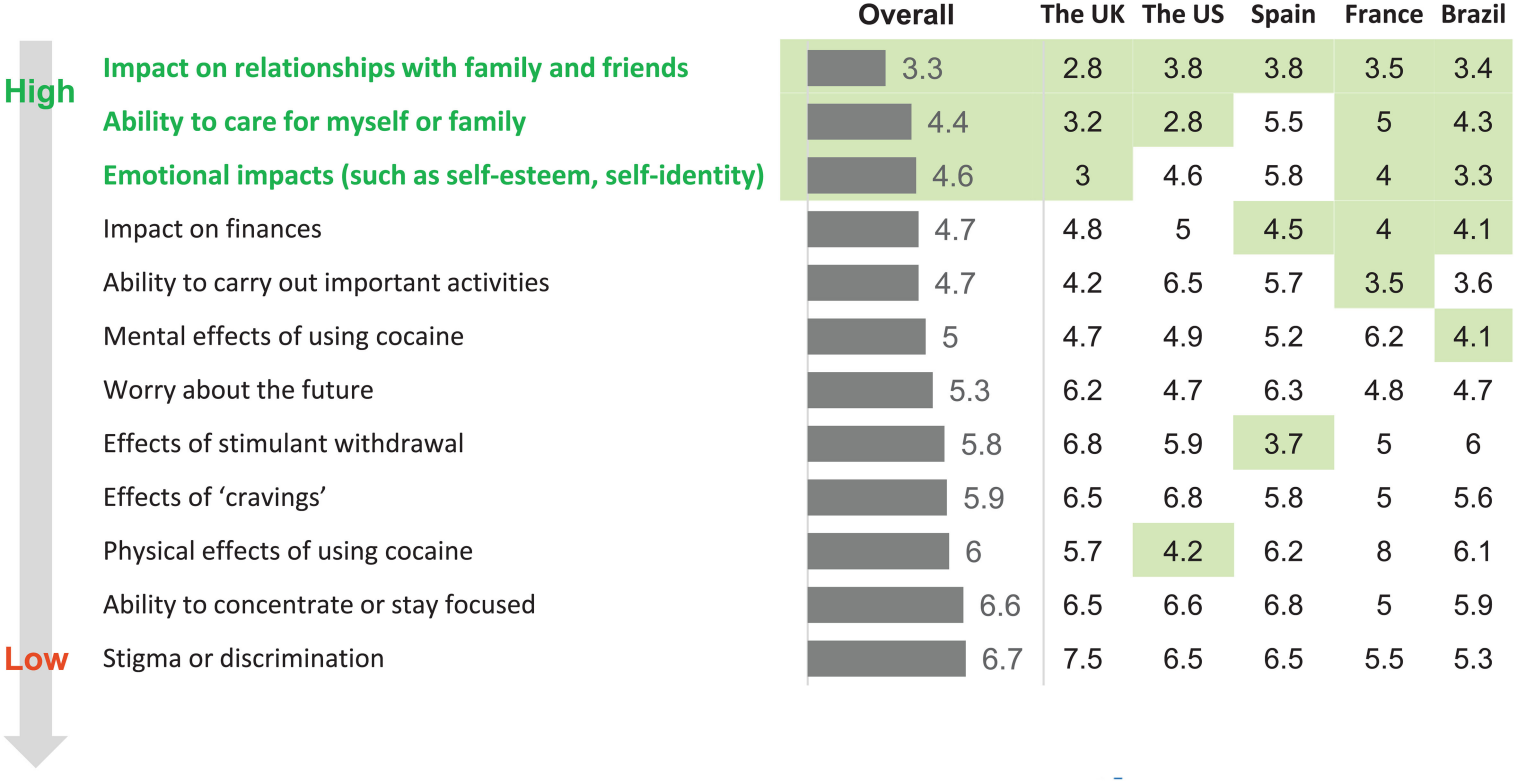
Impact of cocaine use on individuals in cocaine use disorder (CUD) remission.

Individuals in CUD remission felt that cocaine had changed their personality and some felt that it had such a detrimental effect on their relationships that their family members and friends avoided them. For example, a participant in the UK shared, “Impact on my family was the main thing that bothered me, I neglected my kids emotionally, I wasn’t in the right frame of mind to give them what they needed.” They felt shame and embarrassment that caused them to pull away from loved ones. In their opinion, the main concerns of their family members and friends who were close to them included: death from overdose, impact on long-term mental health (depression, mood swings and personality changes), relationship breakdown, becoming estranged, impact on other family members (especially children), trouble with the law, including incarceration, and loss of trust (eg., due to stealing and lying). Individuals in CUD remission reported when they were regularly using cocaine, they were emotionally unavailable, they missed important family events, and showed disinterest in their relationships. This led them to avoid people and they became isolated and lost relationships (friends, parents, siblings, and children). It was shared that “you are in that vicious cycle you don’t take care of [yourself] and tend to push away the people you love.” Yet, individuals reported they did not feel that they had the power to stop cocaine use. ‘Impact on relationships with family and friends’ ranked as the highest impact cocaine had on their life (**Figure 3**); and from the participant’s perspectives, this negative impact was recognized most often retrospectively once they were in recovery.

**Figure 4:**
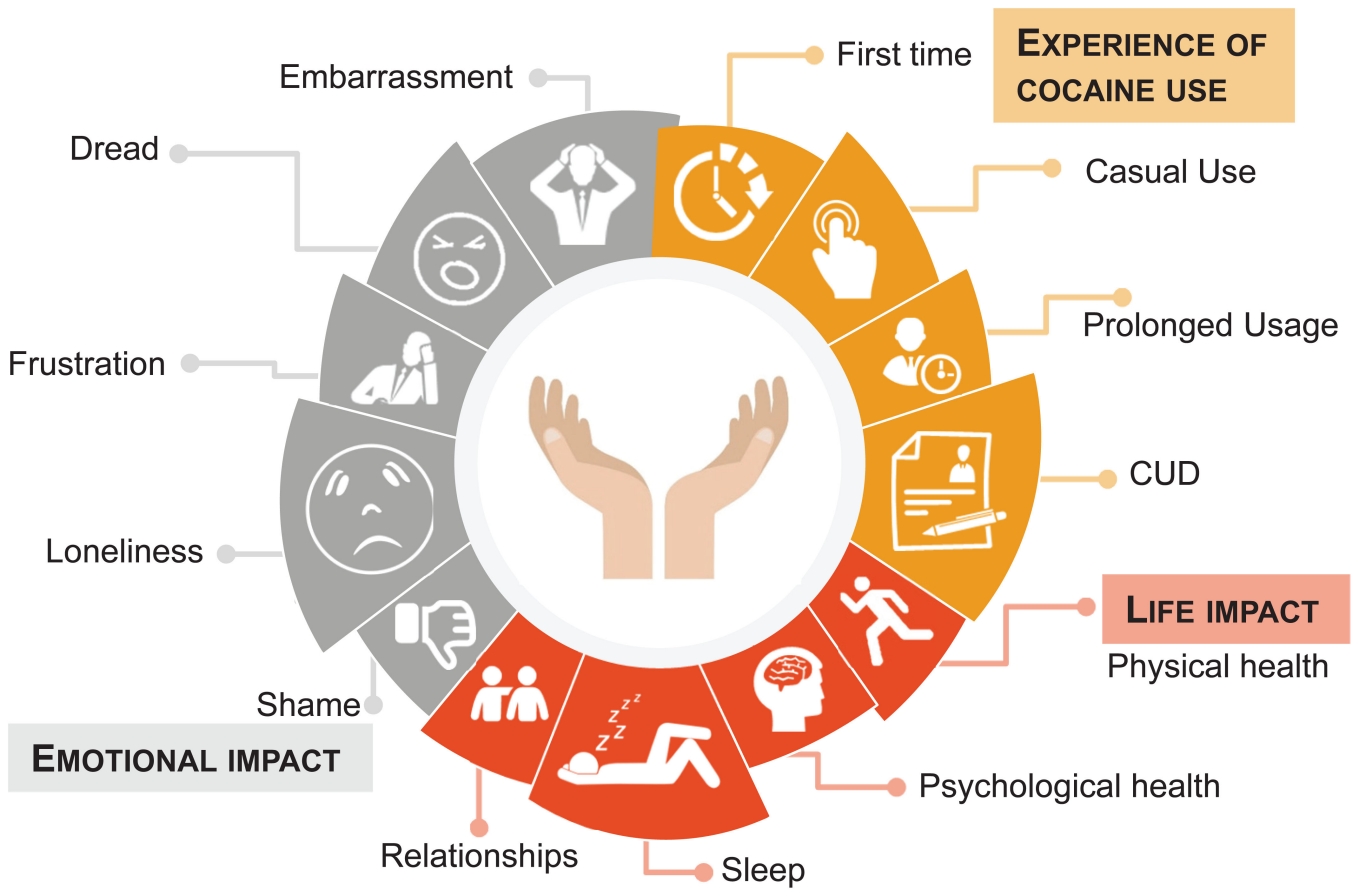
Experiences of cocaine use and its impacts among individuals in cocaine use disorder (CUD) remission.

**Figure 5:**
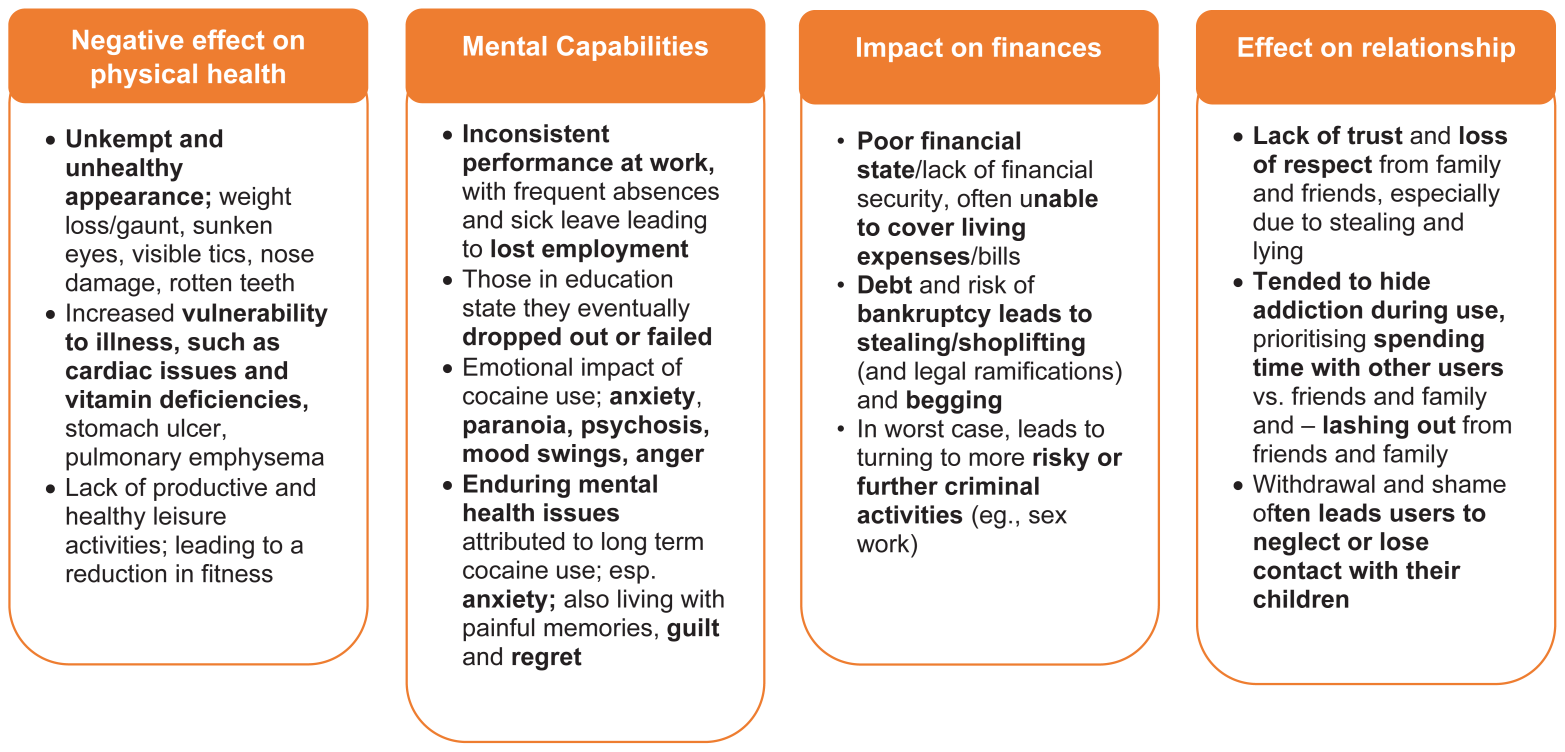
Impact of cocaine use on physical health, mental capabilities, finances, and relationships among individuals in cocaine use disorder (CUD) remission.

There were many experiences and negative impacts of cocaine use among those in CUD remission (**Figure 4**), some of which were immediate, whereas others took years to become fully apparent. Individuals in CUD remission reported that cocaine use had a negative impact on their mental and physical health and affected finances and relationships (**Figure 5**). A participant in the US shared that they “had feelings of hopelessness, worthlessness, and suicidal thoughts constantly” and another participant in Spain shared that “it (cocaine use) was a destructive force and very controlling of me.” The participants felt as if they had prioritized cocaine use over their families and neglected themselves, which led to a decline in their physical health. The main factors leading to a decline in their physical health were reported: poor sleep, fatigue, poor eating habits, lack of exercise, and disregard for risks to health and hygiene. Some of the participants cited they also struggled to care for their dependents, and some had lost custody of their children. All individuals in CUD remission expressed negative psychological effects of cocaine use on their mental health. Cocaine use was often initiated as a form of escape from feelings they did not want to address, and prolonged cocaine use exacerbated these feelings. The most common psychological effects of cocaine use included high levels of anxiety, depression (including suicidal thoughts), low self-esteem, lack of concentration, nervous breakdowns, panic attacks, psychosis, and insomnia.

### 3.3 Supporters

Supporters were considered a close friend or family member of someone who used cocaine regularly. They were all women (n=6) aged 33 to 53 years and all participating from the US (**Table 1c**). Supporters felt powerless, ashamed, betrayed, and surprised when they first learned that their loved one had started to use cocaine. Later, when supporters knew that the individuals they cared for had been using cocaine for a while they felt defeated, helpless, resentment, and were concerned. Supporters felt sad, isolated, and desperately worried about the potential for overdose in individuals with current CUD, as a participant stated: “I hear more and more stories of coke being cut with fentanyl and people overdosing and dying.”

Supporters had some hope for the future to find help, recover, and ‘overcome the demon’. They were extremely involved in helping individuals with current CUD: 5 out of 6 supporters searched for treatments or therapies through various rehabilitation centers, online resources, treatment centers, friends, individuals with prior CUD, and personal doctors. Supporters had a sense of loyalty and family unity, and they were concerned about the consequences of withdrawing their support. They thought that completely reducing or giving up support to individuals with CUD was not an option, but that setting boundaries around their support was acceptable. Some participants shared the importance of supporting their loved one, “Cause she’s family and no matter how I feel about the situation, this is her life … I will continue to support and love her” as well as, “Because I know that if I withdrew my support he might go out and get locked up or hurt.” However, supporters stated they learned to set boundaries with their friend or family member, including actions like not giving the loved one money and attempting to not get overly emotionally involved.

Stress was shared as a major impact on supporter’s lives, with consequences on their mental health (worry, bad nerves, lack of sleep, nightmares, and not taking care of themselves) and physical health. Reported stress and lack of sufficient self-care contributed to physical health problems such as heart attacks, asthma, migraines, and stomach ulcer.

Supporters who accessed help for themselves were satisfied with the support: 4 out of 6 supporters sought help for themselves by using helplines, private therapy with counsellors, programs offered by treatment clinics to friends and family, support offered by 12-step programs, support groups, pastors, recovery groups, online support, social media, and books. The remaining two supporters had not sought help for themselves: one supporter had never considered this as an option and was not aware of anything available; the other supporter had learned about support available through clinics with addiction programs but felt that cost and accessibility were barriers.

### 3.4 Cocaine use disorder journey: CUD remission, current CUD, and supporters

Individuals in CUD remission described their journey to recovery as one with many steps and occasional relapses. From the perspective of individuals in CUD remission and supporters, the main goal was to achieve long-term abstinence from cocaine use; other goals included to stop or reduce cravings, improve physical and mental health, and improve quality of life, including in work and relationships. Individuals in CUD remission indicated that they usually first tried cocaine as teenagers, when heavily influenced by friends. As the cocaine use continued, it became hard to break out of their established social circles. A desire to escape from reality and belong to a group were the common drivers for the first and continued use of cocaine, respectively. For example, a participant in recovery shared that one influence they had for cocaine use sourced from the desire to be part of something: “I wouldn’t say ‘peer pressure’ but the need to belong to a group.” Additional motivations were enhancing performance (mental and physical) and weight loss. A participant in Brazil indicated that, “I met people who got into consuming cocaine just to lose weight and eventually regretted it bitterly.”

Once recovery started, the effect of withdrawal was stated as an ongoing battle to fight cravings. Withdrawal was accompanied by negative feelings of irritability, low confidence, self-hatred, fear, and doubt. Individuals in CUD remission realized that there would always be an on-going effort to assess and manage potential triggers for cravings. To avoid triggers to use cocaine, recovered participants would often distance themselves from friends, places, and activities. Furthermore, those in CUD remission were worried about the risk of future relapse (meaning continued work on recovery takes priority in their lives – often considered a life-long commitment), and they feared that trust and relationships may never be rebuilt in the future.

Cutting down on cocaine use was commonly tried across all groups but ultimately deemed ineffective or did not last for long. Participants shared that their journey to recovery was not straightforward, with many relapses before abstinence from cocaine use was met. The actual timescales to reaching abstinence varied by individual, but once individuals in CUD remission decided to seek help, treatment was initiated quickly within a week. When describing their recovery story, a participant in the US shared the individuals who were helpful in their recovery, “Lots of people have been key in my life: my family, my friends, a psychologist I once had, my sponsors who I have been (with) for years.” During the CUD journey “self-help and reducing cocaine”, “relapse” and “success” were the three stages where individuals spent most of the time. Relapse and re-treatment took as long as 5 to 10 years, although a minority found success the first time they attempted to quit cocaine. A stable period of recovery was as extensive as 20 years, although some individuals in CUD remission indicated they still experienced cravings.

### 3.5 Journey of stopping cocaine use: current CUD and CUD remission

**Figure 6:**
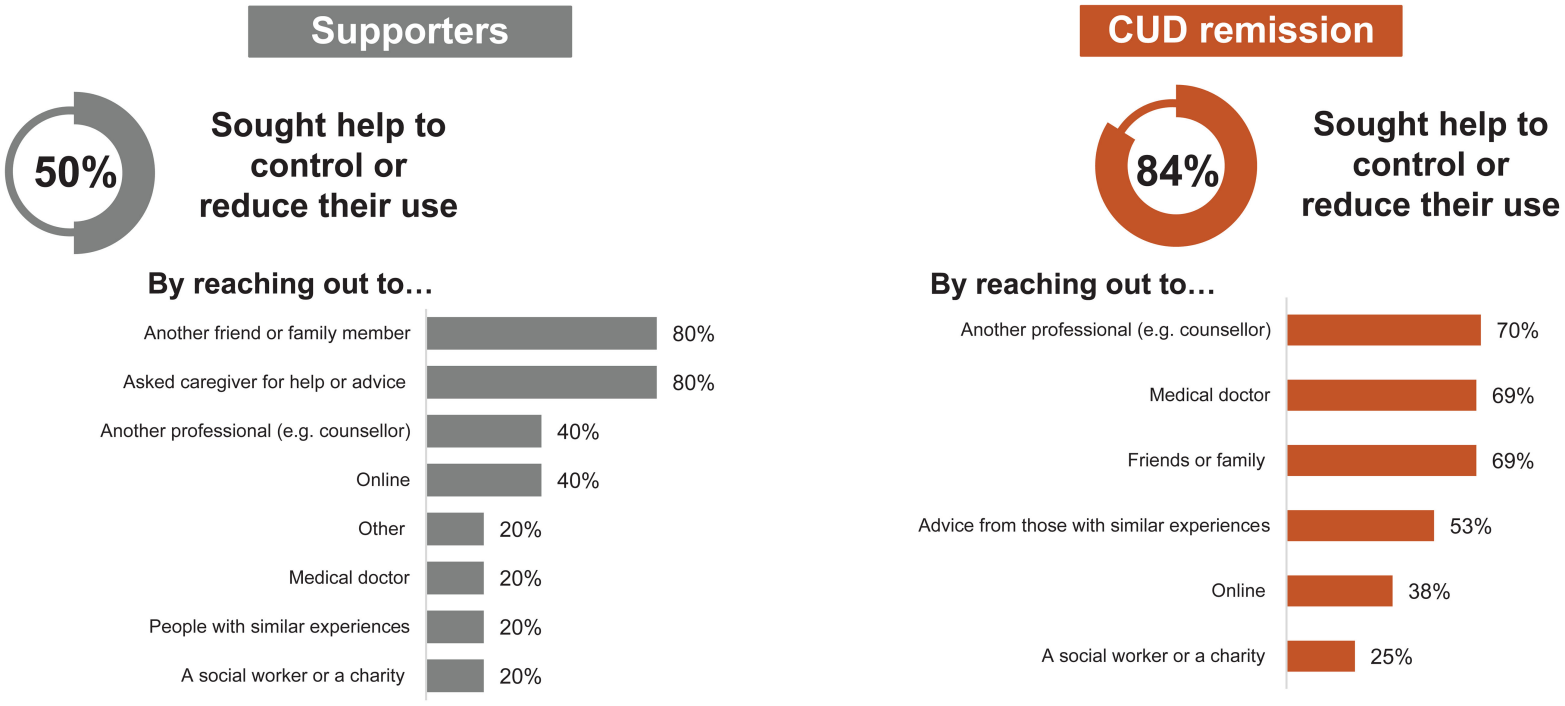
Levels of professional involvement among individuals in cocaine use disorder (CUD) remission and supporters.

Several individuals with current CUD tried to stop using cocaine, however, the participants shared that nothing worked to date to stop their use. Substitution of cocaine with alternative substances (alcohol, cannabis, pain medication) was common, and all individuals with current CUD returned to using cocaine because they reported that cocaine was easily accessible. For example, a participant shared their past plans for substitution, “I’m only gonna smoke pot now, like that was my solution for supporting myself.” Despite having ‘close friends or family members’ as key supporters, individuals with current CUD hesitated to involve them: one participant described they had spoken to their parents and was encouraged to seek help; however, the remaining individuals did not believe that their drug use affected their family members enough to seek their opinion. All individuals with current CUD thought about a ‘healthcare professional intervention’ but most of them had not initiated a conversation with their healthcare professionals (HCPs). A participant stated, “I wanted to go see a doctor, somebody who can help me stop, but then I remember when I will tell him I use these drugs, I don’t know how they will feel about me and they will feel … I feel embarrassed.” Social stigma was cited as the largest barrier in having conversation with their HCPs. However, those who reported speaking to their HCPs were worried that interventions could permanently be on their medical records, and they would be perceived as ‘drug seekers’ in the future. Individuals in CUD remission had higher levels of professional involvement (84%) whereas 50% supporters reported seeking professional help regarding their loved ones CUD (**Figure 6**). From the viewpoint of individuals in CUD remission, they believed it is very difficult to sustain long-term abstinence without professional support. As stated by a participant in Spain, “Abstinence is difficult because you are tempted in social situations (going out, drinking, questions you get asked). Abstinence is very difficult to take on, it requires tremendous effort and constant vigilance.”

Individuals in CUD remission stated that they attempted self-help by trying to control their frequency and amount of cocaine used, compartmentalizing so that cocaine did not infringe on specific areas, and embarking on lifestyle changes (reducing consumption or total abstinence through changed habits) to support reduced cocaine use. A participant in recovery in Spain shared what helped them in reaching recovery, “Quitting cocaine leaves a void that must be filled, and you have to know what to fill it with to be able to feel whole and to not have that void come back again. Discovering nature, natural things, ecological agriculture, gardens, all of that, at least for me, is what worked and is still working.”

Some individuals in CUD remission also shared that when they attempted to reduce use in the past, they felt some positive changes, such as lowering their risk of overdose, improving relationships, and feeling as if they have greater control over their lives. For example, a participant in Brazil shared, “After reducing the use, I was quite quick to notice (the) body and mind getting better.” However, 24% individuals in CUD remission reported that they felt there was no positive change following past attempts at reduced consumption. One participant in the US shared, “I struggled with ‘reducing’. I told myself that I would only use a set amount, but always found a way to rationalize breaking the promise.” Participants felt that the positive gains from reduced use were usually modest, and cocaine use tended to re-escalate to previous levels at some point. Once attempts at reducing use were not successful, participants did share that they thought of abstinence as an option, but there were the following barriers to achieving this: emotional barriers, fear of unpleasant effects of withdrawal (physical and psychological), peer pressure, and feelings of not knowing where to go.

### 3.6 CUD: comorbidities

Participants shared that CUD is associated with many comorbidities, some of which participants felt may drive their cocaine use further, whereas other comorbidities were a consequence of CUD. Participants reported that the existing conditions thought to drive cocaine use included alcohol use, other substance use disorders (e.g., opioid use disorder, amphetamine/methamphetamine use disorder), hyperactivity, migraines, anxiety, depression, and mental health diagnoses (e.g., borderline personality disorder, attention deficit hyperactivity disorder, and human immunodeficiency virus infection). One participant in the UK shared that, “When I was 22, I had a heart attack, I had been out on a 3 day bender, no sleep, no food, just straight cocaine and drinking.” Some health diagnoses were viewed by participants as a consequence of cocaine use including, sinus problems, teeth problems, hepatitis A, B and C, heart disease, hypertension, high blood pressure, rheumatoid arthritis, and cardiomyopathy.

## 4 Discussion

In this study, we used an OBB and focus group methodology to generate insights into the addiction journey and experiences of individuals with CUD (current CUD and CUD remission) and supporters who cared for them. To the best of our knowledge, this was the first study reporting the patient and supporter journey in CUD. Although qualitative research can be complex and nuanced, we have attempted to aggregate and highlight the key challenges and unmet needs participants shared about their experiences using cocaine as well as the feelings and emotions evoked from living with CUD.

Individuals with CUD were from varying socioeconomic groups, but they reported a shared desire for the ‘euphoric high’ that cocaine provided. Individuals with current CUD were still attached this feeling that cocaine provides, whereas those in CUD remission had more negative perceptions about their past cocaine use. Supporters reported they struggle to find a balance between what they perceived as enabling versus supporting their loved one. All individuals in CUD remission stated they hit a personal rock bottom (**Figure 2**) where they realized they needed help to stop their cocaine use and improve their quality of life. Stopping cocaine use was described as largely positive among individuals in CUD remission as they felt they regained control over their lives, reduced anxiety, improved work performance, felt more energized, felt physically healthier and regained financial control. However, the negative consequences included a loss of identity and intense negative emotions such as depression and anxiety.

Continued cocaine use had a negative impact on the mental and physical health of individuals with CUD, their financial stability, and relationships with close family and friends. Feedback about the impact of using cocaine varied within the study groups (**Figure 1**). Individuals with current CUD perceived the impacts of CUD as relatively minor or reversible, especially compared to other drugs, while the financial impact from CUD seem to have the most day-to-day impact. Individuals in CUD remission were more likely to perceive impacts as major and long term, with the negative impact on relationships being the most referenced.

Comprehensively understanding the patient and supporter journey can have key impacts in treatment and intervention design. By understanding the barriers, emotions, and journey to addressing CUD, researchers and healthcare providers can better provide patients with care that meets their needs (12, 13). This can influence a breadth of healthcare planning from as early as creating patient-centered outcomes in clinical trials to designing healthcare settings that ensure anonymity, safety and stigma-free care and support; a major barrier to care cited by this study’s participants.

This study also highlights the integral role supporters have in the recovery of people living with CUD. The results illustrate the impact that caring for someone with CUD may have on the supporters themselves – respondents shared feelings of sadness, isolation and worry for the loved one. This high emotional burden shared by supporters is consistent with other literature (5, 14, 15) on the impact of supporters for those with SUD. Based on the participant’s responses, addiction not only impacts the patient, but their supporters. These individuals can be critical lines of support for those living with addiction as they navigate how to reach and sustain recovery. It is crucial that healthcare interventions acknowledge the importance of supporters and include these loved ones as an active part of a comprehensive medical response to addiction and recovery (14, 15).

One of the key strengths of the methodology used in this study was to maintain patient anonymity on the open virtual forum, leading to interactive discussions and responses from participants while dealing with sensitive topics. There are several limitations in our study. First, there was a limited geographical distribution of patients across the groups. Individuals with current CUD and supporters were from the US only, because of cultural differences toward substance use and their willingness to talk about it. This led to a smaller number of supporters and people living with CUD that participated in the study, which may lead to bias in the results. Second, because of the short duration of the study and the small sample size of participants the results may not be generalizable to the larger population. Third, some participants (6% of the individuals in CUD remission) had no diagnosis consistent with CUD, although they did have at least five criteria of SUD per DSM-5 criteria. Furthermore, there were some questions that varied between the OBB and the focus group due to the limitations of the format for the focus group.

## 5 Conclusion

The OBB and focus group provided the perspectives of patients and supporters, as well as valuable insights into their experiences using cocaine. The robust and meaningful data generated from this study enable assessment of the knowledge gap and provide insights into this under-served and growing population. Understanding the patient journey with emphasis on their experiences and needs will guide drug development. Further, more work is needed to ensure patient experience data is systematically used to help inform drug development, regulatory decision-making, and to improve the quality of healthcare.

## Data availability statement

The original contributions presented in the study are included in the article/**Supplementary Material**. Further inquiries can be directed to the corresponding author.

## Ethics statement

This study was conducted in accordance with European Pharmaceutical Market Research Association (EphMRA) guidelines (16) which state that Ethics committee approval is not required if study met the criteria of market research. Therefore, Institutional Review Board (IRB) approval was not sought for this market research study. All procedures involving human participants were performed in accordance with the Declaration of Helsinki and the guidelines for Good Epidemiology Practice. Written informed consent to participate in this study was provided by the patients/participants. Participants had the right to withdraw at any time during the study.

## Author contributions

DL: Conceptualization, writing the original draft; visualization, supervision, funding acquisition, reviewing and editing all the subsequent drafts. BG-M: Conceptualization, methodology, writing the original draft; visualization, supervision, funding acquisition, reviewing and editing all the subsequent drafts. SM: Conceptualization, methodology, validation, visualization, writing the original draft, reviewing and editing all the subsequent drafts. KW: Conceptualization, methodology, validation, visualization, project administration, writing the original draft, reviewing and editing all the subsequent drafts. VM: Visualization, formal analysis, reviewing and editing all the subsequent drafts. PB: Visualization, formal analysis, reviewing and editing all the subsequent drafts. BK: Visualization, writing the original draft; reviewing and editing all the subsequent drafts. AK: Writing the original draft, reviewing and editing all the subsequent drafts. All authors have approved the final article.
