# Supplementary material for: Understanding the patient and supporter journey in cocaine use disorder
Source: Front Psychiatry. 2024 Apr 10;15:1230626. doi: 10.3389/fpsyt.2024.1230626 (PMC11040082; doi:10.3389/fpsyt.2024.1230626)
Supplement: Supplementary file 1 [file Presentation_1.pdf]

Appendix 1

Study procedures

Online bulletin board (OBB) forum: After completion of the screening questionnaire, individuals in cocaine use disorder (CUD) recovery and supporters logged into the OBB forum and responded to questions posed by a trained moderator (see Supplementary material 3). After the completion of the OBB, there was no additional follow-up for participants. Participants were encouraged to engage in anonymous discussions with other participants and exchange views and experiences. They could log onto the OBB platform and complete the questions at a time that suited them. For the OBB, unless otherwise stated, all responses were visible to other participants in this research. At times the moderator invited participant to review and comment on other participants' answers or posts via email notification. In most of these cases, a participant was not able to see or comment on others' answers until they had posted a reply for the activity that day. When a participant wanted to respond to the moderators or to anyone else, they hit the "Reply" button on the question or comment they were responding to. If the participant wanted to keep a response private for any reason, the participant sent emails or private messages to the moderator through the messaging facility within the platform. One section of the board was private to individual respondents (i.e., section on comorbidities).

Virtual focus group discussion: Individuals with current CUD were requested to join a virtual structured focus group discussion for 90 minutes in a single day hosted by a trained moderator. After the completion of the OBB, there was no additional follow-up for participants. Participants discussed between themselves during the live session (conducted via audio only and

not video) and were able to take a break at any time. All recordings of the focus group were destroyed after transcription.

Appendix 2

Anonymity of data

The study was conducted according to all national data protection laws and relevant industry guidelines, including European Society for Opinion and Marketing Research (ESOMAR), European Pharmaceutical Market Research Association (EphMRA), and British Healthcare Business Intelligence Association (BHBIA; UK only), and all other relevant national codes of conduct.

The study was designed to help participants to maintain compliance with local data protection and privacy. Anonymity was maintained among other participants on the forum, and any identifiable data to the third parties were kept confidential. The anonymity of the participants was protected during the study by using an ‘anonymous ID’. The name, location, or any other identifying information of the participant was not used during the study. Participants were requested not to mention any identifiable information during the online community and were requested not to upload any identifiable images or videos to ensure their anonymity. If any participant mentioned identifiable information, it was removed from the OBB by a moderator, allowing participants to remain anonymous. For the live focus group that included participants with current CUD, pseudonyms were used during conversation. A confidential online platform (FocusVision InterVu, USA) was used to ensure participants real names and/or locations were not displayed once they logged in.

Appendix 3

Screening questionnaire (attachment)
