# Supplementary material for: Understanding the patient and supporter journey in cocaine use disorder
Source: Front Psychiatry. 2024 Apr 10;15:1230626. doi: 10.3389/fpsyt.2024.1230626 (PMC11040082; doi:10.3389/fpsyt.2024.1230626)
Supplement: Supplementary file 2 [file Presentation_2.pdf]

Supplementary Material

Understanding the patient and supporter journey in cocaine use disorder

Denise Leclair,* Katherine M. Waye, Baltazar Gomez-Mancilla, Brian D. Kiluk, Ananda Krishna Karanam, Partha S. Banerjee, Velusamy Shanmuganathan Muthusamy, Suzanne Maahs

*Correspondence: Denise Leclair denise.leclair@novartis.com

Contents **Page number**

Supplementary material 1	Methodology	1
Supplementary material 2	Screeners	2
Supplementary material 3	Task flow	20

Supplementary material 1: Methodology

A mix of recruitment methods was used, including contacting patients on market research panels, social media advertising in relevant special interest groups, and referrals from volunteers at rehabilitation clinics. Shopping vouchers with cash equivalent were paid to the participants at the end of the study.

For the online bulletin board (OBB), the full amount of time required for participation in this research was 75 minutes – 60 minutes of participation in the board and 15 minutes of onboarding time. For individuals with current cocaine use disorder (CUD) taking part in

the focus group, the full amount of time required was 105 minutes, which consisted of a virtual group discussion lasting 90 minutes with other people who use cocaine and 15 minutes of onboarding time.

This text appeared at the beginning of the OBB and was made clear to the respondents at the screening stage:

> "Your anonymity is protected during this study by using an anonymous ID. Your name, location or any other identifying information is not used during the study. Please do not mention any identifiable information during the online community (such as your full name or contact details) and please do not upload any identifiable images or videos of yourself or anyone else to ensure your anonymity. If you do mention any identifiable information, this will be removed from the board by the moderator, to allow you to remain anonymous."

> "Unless otherwise stated, all responses will be publicly visible to other participants in this research. We may also invite you at times to review and comment on other participants' answers or 'posts' via email notification. Our discussion is more interesting when everyone takes part in the dialogue and builds upon one another's answers. Please note that, in most of these cases, you will not be able to see or comment on others' answers until you have posted a reply yourself for the activity that day. When you want to respond to the moderators or to anyone else, just hit the "Reply" button on the question or comment you are responding to. If you want to keep any response private for any reason, please private message the board moderator through the messaging facility within the platform or email us at: …."

One section of the board (the section on comorbidities) was private to individual respondents, and this was clearly stated in this section. For the focus group with individuals with current CUD, participants had discussions between themselves during the live session. Pseudonyms were used in the conversations. A special online platform (InterVu, FocusVision, USA) was used to ensure real respondent names or locations were not displayed once respondents were logged in. There were no follow-up questions for those participating in the OBB or the focus group.

For the bulletin board and the focus group, the main output was a transcript of the discussion. The bulletin board generated some additional outputs, such as images and responses to closed questions (e.g., agreement with statements or polling) which were analyzed alongside the transcripts. Anonymized data underwent thematic analysis. Transcripts were reviewed iteratively by several project team members to identify and examine key themes and patterns in the interviews both within and across markets and patient demographics.

Project team members then came together to agree on the research findings. Recordings were only for the purpose of transcription and were destroyed after completion of the study.

Supplementary material 2: Screeners

SCREENING QUESTIONS – INDIVIDUALS IN CUD REMISSION

S1.	Are you, or any member of your household, employed by the pharmaceutical industry on a full-time or consultancy basis, or is any member of your household employed by a communication or advertising industry related to pharmaceutical products?<br><br>Yes ....1 CLOSE<br>No 2
S2.	Which age bracket do you fall into?<br><br><18 ....1 CLOSE<br>18-29 .... 2<br>30-39 .... 3<br>40-49 .... 4<br>50-59 .... 5<br>60+.................... 6
S3.	Have you been diagnosed by a physician with any of these conditions?<br>Migraine .... 1<br>Hypertension or high blood pressure 2

	Anxiety Disorders .... 3<br>Diabetes........ 4<br>Substance Use Disorder .... 5<br>Cocaine Use Disorder .... 6<br>Opioid Use Disorder .... 7<br>Amphetamines/Methamphetamines Use Disorder .... 8<br>Depression Disorders .... 9<br>Rheumatoid Arthritis........ 10<br>Crohn's disease .... 11<br>Alcohol Abuse/Alcoholism .... 12<br>Other .... 13<br><br>MUST SELECT CODE 5 OR 6 OR SAY YES TO 5 OR MORE STATEMENTS IN S13
S4.	In addition, have you even been diagnosed with any of the following?<br><br>Schizophrenia .... 1 CLOSE<br>Bipolar Disorder........ 2 CLOSE<br>Borderline Personality Disorder .... 3 CLOSE<br>Dementia or Alzheimer's Disease........ 4 CLOSE<br>Post-Traumatic Stress Disorder (PTSD) .... 5 CLOSE<br>Attention Deficit Disorders .... 6<br>Other: _________ .... 7<br>None of the above .... 8 CONTINUE

S5	Which of these statements best reflects your relationship with stimulant(s) or any other illicit drug(s)?<br><br>In the past I used to regularly use a stimulant(s) or any other illicit drug(s), but I currently do not take any stimulant(s) or any other illicit drug(s) .... 1<br>I currently regularly use a stimulant(s) or any other illicit drug(s).......... 2 CLOSE<br>I have never used stimulant(s) or any other illicit drug(s) .... 3 CLOSE
S6	Which stimulant(s) or illicit drug(s) have you regularly used in the past?<br>(An illicit drug is a drug that is not legally permitted or authorized.)<br>____<br><br>Respondent must spontaneously state ‘**crack cocaine’ or ‘cocaine’** to screen in
S7	How frequently did you use cocaine in the past?<br><br>More than once a day .... 1<br>Daily .... 2<br>Weekly .... 3<br>Monthly .... 4<br>Only on occasion .... 5<br>Other 6

MUST SELECT CODE 1 - 4

S8

Which of the following treatments have you used in the past or are currently using in relation to your cocaine use (if any)

**Treatment**	**Currently using**	**Have used in the past**
a) Counselling/psychological support		
b) In-patient detox/ rehabilitation		
c) Cognitive-Behavioural Therapy (CBT)		
d) 12 Step program(mes) and peer support group (e.g. Narcotics Anonymous)		
e) Individual and/or family therapy		
f) Complementary/alternative medicine		
g) Other		
h) None of the above		

Please ensure natural fallout across the different therapies (a-f) either current or previous and 'none of the above' (h)

S9

Which of the following statements describe your past relationship with cocaine?

		Y/N
Loss of Control	I took cocaine in larger amounts or over a longer period of time than I intended	
	I had a persistent desire or unsuccessful attempts to cut down or control my cocaine use	
	I experienced cravings or a strong desire or urge to use cocaine	

			I spent a great deal of time taking part in in activities to obtain, use, or recover from the effects of cocaine	
		Risky Use	I recurrently used cocaine in situations where it was physically hazardous	
			I continued using cocaine despite physical and/or psychological problem(s) which were likely to have been caused or exacerbated by cocaine	
		Social Impairment	Recurrent cocaine use resulted in a failure to fulfill major role obligations at work, school or home	
			I continued using cocaine use despite having persistent or recurrent social and/or interpersonal problems caused or exacerbated by the effects of cocaine	
			I gave up or reduced my time spent in important social, occupational, or recreational activities because of my cocaine use	
		Pharmacological Effects	I experienced a significant increase in tolerance to cocaine whilst I was regularly using it	
			I experienced withdrawal symptoms when I stopped regularly using cocaine	
	MUST SAY YES TO 5 OR MORE STATEMENTS OR REPORT HAVING AN OFFICIAL DIAGNOSIS OF COCAINE USE DISORDER OR SUBSTANCE USE DISORDER IN S3			
S10	Are you comfortable discussing cocaine use and remission at length via on online bulletin board where **your anonymized (no link back to you that will make you identifiable) responses will be shared with other individuals**?<br>Please note, your real name will not be revealed on the bulletin board, but some of your anonymized responses will be visible to other participants on the board			

	Yes .... 1<br>No .... 2 CLOSE
S11	Do you have regular internet access via a desktop computer / laptop/ tablet in order to participate in an online bulletin board?<br>Yes .... 1<br>No .... 2 CLOSE
	Only ask the D questions if the respondents screens in otherwise, terminate the call.
D1	What is your gender?<br>Male.. 1<br>Female .... 2<br>Prefer not to answer .... 3<br>Neither of these 4
D2	What is the highest degree or level of education you have completed?<br>High school graduate, diploma or the equivalent.. 1<br>Bachelor's degree.. 2<br>Master's degree .... 3<br>Professional degree.. 4<br>Doctorate degree 5

	Other (please specify) .... 6<br>None of the above.......... 7<br>Prefer not to say.......... 8<br><br>Recruitment note: If 'other' please make a note
D3	What is your or your household's annual income level (before tax)?<br><br>Less than $24,999.......... 1<br>$25,000 - $49,999 .... 2<br>$50,000 - $74,999 .... 3<br>$75,000 - $99,999 .... 4<br>$100,000 +.......... 5<br>Prefer not to say.......... 6
D4	Which setting best describes where you live?<br><br>Urban.......... 1<br>Suburban .... 2<br>Rural 3
D5	US ONLY: Which state do you reside in?<br><br>_____________________

D6	In relation to cocaine/ crack cocaine (read out as appropriate) **how long have you not used it for and/or been in remission with no relapses?**<br>______________ Years ______________Months
D7	US ONLY: What is your insurance type?<br>Medicare .... 1<br>Medicaid .... 2<br>Private health plan offered through an employer or other organization.......... 3<br>Private health plan or insurance that you purchased yourself......................... 4<br>Other (please specify) .... 5<br>I have no health insurance .... 6<br>Don't know/unsure .... 7<br>I prefer not to answer 8

SCREENING QUESTIONS – Supporter

S1.	Are you, or any member of your household, employed by the pharmaceutical industry on a full-time or consultancy basis, or is any member of your household employed by a communication or advertising industry related to pharmaceutical products?<br><br>Yes........................ 1 CLOSE<br>No........................ 2
S2.	What is your gender?

	Male .... 1<br>Female .... 2<br>Prefer not to answer .... 3<br>Neither of these 4
S3.	Which age bracket do you fall into?<br><br><18........ 1 CLOSE<br>18-29 .... 2<br>30-39 .... 3<br>40-49 .... 4<br>50-59 .... 5<br>60+ 6
S4.	Are you a close friend or family member of someone who regularly uses a stimulant(s) or any other illicit drug(s)<br>(A stimulant is a drug that quickly increases your alertness and energy. Common stimulants are crack, cocaine and speed. An illicit drug is a drug that is not legally permitted or authorized for use)<br>Yes .... 1<br>No .... 2 CLOSE
S5.	Which of these statements best reflects your friend or family member's relationship with stimulant(s) or any other illicit drug(s)?

	In the past they used to regularly use a stimulant(s) or any other illicit drug(s), but currently do not take any stimulant(s) or any other illicit drug(s) ....1 CLOSE<br>They currently regularly use a stimulant(s) or any other illicit drug(s) 2
S6.	Which stimulant(s) or illicit drug(s) do they regularly use?<br>____<br>Respondent must spontaneously state '**crack cocaine' or 'cocaine'** to screen in
S7.	How frequently does your friend or family member use cocaine?<br>More than once a day.......................................1<br>Daily ....2<br>Weekly ....3<br>Monthly ....4<br>Only on occasion.......................................5 CLOSE<br>Other ....6 CLOSE<br>Unknown.......................................7 CLOSE<br>MUST SELECT CODE 1 -4 – if other please consult Adelphi for advice
S8.	Which of the following statements describe your friend or family member's relationship with cocaine?

		Y/N
Loss of Control	They take cocaine in larger amounts or over a longer period of time than they intend	
	They have a persistent desire or unsuccessful attempts to cut down or control their cocaine use	
	They experience cravings or a strong desire or urge to use cocaine	
	They spend a great deal of time taking part in in activities to obtain or use, or recover from the effects of cocaine	
Risky Use	They recurrently use cocaine in situations where it could physically harm them	
	They continue using cocaine despite physical and/or psychological problem(s) which were likely to have been caused or exacerbated by cocaine	
Social Impairment	Their cocaine use results in a failure to fulfill major role obligations at work, school or home	
	They continue using cocaine despite having persistent or recurrent social and/or interpersonal problems caused or exacerbated by the effects of cocaine	
	They gave up or reduced their time spent in important social, occupational, or recreational activities because of their cocaine use	
Pharm acolog	They have experienced a significant increase in tolerance to cocaine when they are regularly using it	

		They experience withdrawal symptoms when they stop regularly using cocaine	

MUST SAY YES TO 5 OR MORE STATEMENTS OR REPORT HAVING AN OFFICIAL DIAGNOSIS OF COCAINE USE DISORDER OR SUBSTANCE USE DISORDER IN S9.

S9.	Has your friend or family member been diagnosed by a physician with any of these conditions?<br>Migraine....1<br>Hypertension or high blood pressure....2<br>Anxiety Disorders....3<br>Diabetes....4<br>Substance Use Disorder....5<br>Cocaine Use Disorder....6<br>Opioid Use Disorder....7<br>Amphetamines/Methamphetamines Use Disorder....8<br>Depression Disorders....9<br>Rheumatoid Arthritis....10<br>Crohn's disease....11<br>Alcohol Abuse/Alcoholism....12<br>Other....13<br>I don't know....14<br><br>MUST SELECT CODE 5 OR 6 OR SAY YES TO 5 OR MORE STATEMENTS @ S8

S10.	In addition, have they been diagnosed with any of the following?
	Schizophrenia ....1 CLOSE
	Bipolar Disorder ....2 CLOSE
	Borderline Personality Disorder ....3 CLOSE
	Dementia or Alzheimer's Disease ....4 CLOSE
	Post-Traumatic Stress Disorder (PTSD) ....5 CLOSE
	Attention Deficit Disorders 6
	Other: ________ 7
	None of the above ....8 .... CONTINUE
	Don't know ....8 .... CONTINUE

S11. Which of the following treatments is your friend or family member currently undergoing in relation to their cocaine use (if any)

**Treatment**	**Currently using**	**Have used in the past**
i) Counselling/psychological support		
j) In-patient detox/rehabilitation		
k) Cognitive-Behavioral Therapy (CBT)		
l) 12 Step program(s) and peer support group (e.g. Narcotics Anonymous)		
m) Individual and/or family therapy		
n) Complementary/alternative medicine		
o) Other		
p) None of the above		
q) I don't know		

S12. What is your relationship to your friend or family member who regularly uses cocaine?

Partner/Spouse........1
Father........2
Mother........3
Sponsor........4
Son........5
Daughter........6
Friend........7
Other relative (please specify)........8
Other (please specify)........9

S13.	Do you feel you have a **close enough connection** to the person to be able to discuss their cocaine use in detail including the impact on their lives and any therapies they have tried?<br><br>Yes ....1<br>No..2 CLOSE
S14.	a) Are you **comfortable** discussing your friend or family member's cocaine use at length via on online bulletin board where your anonymized (no link back to you that will make you or the friend or family member who uses cocaine identifiable) responses will be shared with other individuals?<br><br>Please note, your real name or the name of the person you care for will not be revealed on the bulletin board, but some of your anonymized responses to questions and/ or task activities outlined by the moderator will be visible to other participants on the board<br><br>Yes ....1<br>No ....2 CLOSE<br><br>b) Are you **comfortable** discussing how your friend or family member's cocaine use impacts **your life** and **your** personal relationship with them?

	Yes ....1<br>No ....2 CLOSE
S15.	In relation to cocaine/crack cocaine (read out as appropriate) approximately **how long has** your friend or family member **used it for?**<br>______________ Years ______________Months
S16.	Does your friend or family member have a perceived willingness to quit using cocaine/crack cocaine (read out as appropriate)<br>Yes..1<br>No..2
S17	Do you have regular internet access with a desktop computer/laptop/ tablet to participate in an online bulletin board?<br>Yes ....1<br>No ....2 CLOSE
S18	a) What is the highest degree or level of education your friend or family member has completed?<br>High school graduate, diploma or the equivalent ....1<br>Bachelor's degree 2

	Master's degree...3<br>Professional degree ....4<br>Doctorate degree...5<br>Other (please specify).....................................6<br>None of the above ....7<br>Prefer not to say ....8<br>Unsure...9<br><br>Recruitment note: If 'other' please make a note<br><br>b) What is the highest degree or level of education you have completed?<br><br>High school graduate, diploma or the equivalent ....1<br>Bachelor's degree ....2<br>Master's degree...3<br>Professional degree ....4<br>Doctorate degree...5<br>Other (please specify).....................................6<br>None of the above ....7<br>Prefer not to say ....8<br>Unsure...9

S19	Which setting best describes where your friend or family member lives?<br><br>Urban .... 1<br>Suburban.. 2<br>Rural.. 3
S20	Which state does your friend or family member reside in?<br>____________________
S21	What is your friend or family member's living situation?<br>They live on their own full time .... 1<br>They live with me for some period during the week .... 2<br>They live with me full time..3<br>They live with others 4
S22	Do you know if your friend or family member has medical insurance or not? If so, which type of medical insurance do they have?<br>Medicare .... 1<br>Medicaid .... 2<br>Private health plan offered through an employer or other organization .... 3<br>Private health plan or insurance that they purchased themselves..................... 4<br>Other (please specify).. 5<br>They have no health insurance.. 6<br>Don't know/unsure.. 7

	I prefer not to answer...8<br>RECRUIT MIX

Supplementary material 3: Task flow: Supports

Day 1 Task

Thank you for joining today.

Today we will ask you to introduce yourself and tell us a bit more about your experiences being close to somebody who uses cocaine. We understand this can be a sensitive topic so please be reassured we are not here to judge or criticize anyone, we genuinely want to hear your real-life experiences.

A. Please tell us a little bit more about yourself e.g. what you do, how you like to spend your time? Please do not reveal any information that could help identify yourself or anyone else.

B. <display images> Thinking of your friend or family member that uses cocaine, which images do you think best describe their feelings about cocaine?

   You can also upload your own image or photo but please remember to not upload anything which may identify you or anybody else

   a. Why have you chosen this specific image(s)?

C. <display images> Which images best describe your feelings about cocaine?

   You can also upload your own image or photo but please remember to not upload anything which may identify you or anybody else

   b. Why have you chosen this specific image(s)?

D. Please can you fill in the blanks to help us understand your emotions about being close to someone who uses cocaine? Please use whatever words fit your experience best.

   i. When I first found out they started to use cocaine, I felt ___________
   ii. Now that they have been using cocaine for a while, I feel ___________
   iii. At the moment I feel ____________ about their cocaine use. My life these days is ____________
   iv. In the future I hope that ____________________

E. Please could you briefly describe your relationship with the person in your life who uses cocaine?

   i. How long have you known the person in your life who uses cocaine?
   ii. What is your relationship with them?
   iii. How regularly do you see them, or how often do you keep in touch?

F. What type of support do you provide to your friend or family member who uses cocaine?

   Practical support I provide:

   Emotional support I provide:

   - Why do you say that?
   - Please explain more

   i. Who else, if anyone, also supports them?

G. On a day to day basis, in what ways do you support your friend or family member who uses cocaine?

   - Why do you say that?
   - Please explain more

H. How did their cocaine use change over time?

- Why do you say that?
- Please explain more

Thank you! Please click 'Next' to continue.

Thank you. That's it for this exercise. When you click 'submit' your responses will be placed on the group discussion board

Then go to 'Discussion Board for Day 1', now available on your study dashboard. Please feel free to come back at any time to see what other members of the group have written and engage in further discussion.

Note for reviewer:

All questions for the Day must be answered before they can proceed to the Discussion Board

The Discussion Board is where respondents are able to interact with each other and all responses written on here are displayed to all. Moderators will be able to post additional Qs for all to see here and/or drag individual's answers from the Day questions to the board for all to see, discuss and react to.

Day 2 Task

Thank you for joining today.

Reminder: please do not mention any details that would incriminate you or anyone else, including any names or other specific details that might identify someone.

This task is focused on understanding in a bit more detail the impact of cocaine use, including physical and emotional wellbeing.

Thinking about your friend or family member you support who is currently using cocaine

A. Out of the following, what do you think have been the most significant impacts of cocaine use on their life [please drag and drop in order of the level of impact or discard if not relevant]
    a. Physical effects of using cocaine (such as rapid or irregular heart rate, difficulty breathing, increased blood pressure, stomach pain)
    b. Mental effects of using cocaine (such as paranoia, confusion, hallucinations)
    c. Ability to carry out important activities (such as work, school, sports, hobbies)
    d. Ability to care for themselves or family
    e. Ability to concentrate or stay focused
    f. Impact on relationships with family and friends
    g. Effects of stimulant withdrawal (such as fatigue, depression, inability to focus)
    h. Stigma or discrimination
    i. Effects of ‘cravings’
    j. Worry about the future (such as relapse, overdose)
    k. Emotional impacts (such as self-esteem, self-identity)
    l. Impact on finances
    m. Other impacts not mentioned [please fill in]

B. Please can you share a bit more about any impact their cocaine use has had on their relationships with family and friends?

- Please explain more
- What makes you say that?

C. Please can you share more about any impacts their cocaine use has had on their physical health?

D. Please can you share more about any impacts their cocaine use has had on their mental health?
    - Please explain more
    - What makes you say that?

E. How does their cocaine use impact your own life?

F. On a scale of 1 – 7, where 1 is no impact at all and 7 is significant impact, how has their cocaine use impacted your physical health? (physical health could include, but not limited to, tiredness, injury, stress related illnesses)
    a. Why did you select that number?
    - Please explain more
    - What makes you say that?

G. On a scale of 1 – 7, where 1 is no impact at all and 7 is significant impact, how has their cocaine use impacted your mental health? (mental health could include, but not limited to, stress, anxiety, insomnia)
    a. Why did you select that number?
    - Please explain more
    - What makes you say that?

H. Can you please share with us any personal health impacts you've had because of supporting someone who uses cocaine?

I. If they ever decreased their cocaine use, what positive changes do you hope to see in their life? Please provide an example(s)
    - Please explain more

Thank you! Please click 'Next' to continue.

Thank you. That's it for this exercise. When you click 'submit' your responses will be placed on the group discussion board.

Then go to 'Discussion Board for Day 2', now available on your study dashboard. Please feel free to come back at any time to see what other members of the group have written and engage in further discussion.

Day 3 Task

Welcome back and thank you for completing Day 2.

Reminder: please do not mention any details that would incriminate you or anyone else, including any names or other specific details that might identify someone.

Today we'd like to understand a bit more about social and support networks for you and your friend or family member you support.

A. In terms of their cocaine use, who or what has a positive impact on their lives?
   - Please explain more
   - What makes you say that?

B. In terms of their cocaine use, who or what had a negative impact on their lives?
   - Please explain more
   - What makes you say that?

C. How open are they about discussing their cocaine use?
   a. With you or with other people?

D. Think about the times they used the most amount of cocaine, what impact did this have on you at that time?
   - Please explain more
   - What makes you say that?
      i. What impact did this have on your relationship with them?
   - Please explain more
   - What makes you say that?
      ii. Was their relationship with other adults affected?

1. Which relationships?
2. How were they impacted?
- Please explain more
- What makes you say that?

E. Think about the times they reduced or stopped using cocaine, what impact did this have on you at the time?
- Please explain more
- What makes you say that?

i. What impact did this have on your relationship with them?
- Please explain more
- What makes you say that?

ii. Was their relationship with other adults affected?

1. Which relationships?
2. How were they impacted?
- Please explain more
- What makes you say that?

F. What are your biggest concerns about their cocaine use?
- Please explain more

a. Why does this concern you?

G. To what extent have you thought about reducing or giving up support for this person?

Please indicate on the below scale where 1 indicates you have never considered giving up support for this person, and 7 indicates you regularly consider giving up support for this person

a. If you feel comfortable, please could you explain why you selected that number?

H. Are you aware of any support available for caregivers of people who use cocaine, such as yourself?

a. Who provides this support?
b. What type of support does it offer?
c. What type of support would you like to receive?
d. Where did you find out about this support?
e. Was it easy to find?
f. Was it easy to access?
   - Please explain more

Programmer: insert emoji pin board exercise

J. Thinking about the **help available** to people like yourself who are supporting someone who uses cocaine please select an emoji which best describes your level of satisfaction with the help available. Why have you selected this emoji?
   - Please explain more

Thank you! Please click 'Next' to continue.

Thank you. That's it for this exercise. When you click 'submit' your responses will be placed on the group discussion board.

Then go to 'Discussion Board for Day 3', now available on your study dashboard. Please feel free to come back at any time to see what other members of the group have written and engage in further discussion.

Day 4 TASK

Welcome back and thank you for completing Day 3.

Reminder: please do not mention any details that would incriminate you or anyone else, including any names or other specific details that might identify someone.

Today we are going to focus on the health of your friend or family member who uses cocaine, how they may look for help or advice for their cocaine/substance use and, if this happened, their journey to a cocaine or substance use disorder diagnosis.

K. Firstly, I'd like to ask about any health diagnoses the friend or family member you support has received from a doctor. This part of our discussion is private and will not be shared with the group. If known, please can you indicate if they have ever been diagnosed with any of the following:

- Migraine
- Hypertension or high blood pressure
- Anxiety disorder
- Diabetes
- Rheumatoid Arthritis
- Crohn's Disease
- ADHD
- Hepatitis C
- HIV
- Opioid use disorder
- Alcohol use disorder
- Depression
- Other [please fill in]

(Continuing privately, your response to this question will also not be shared with the wider group) Or if they have ever been hospitalized because of:

- Withdrawal symptoms from their cocaine use
- Overdose from their cocaine use

L. Do they take any medication for any of the above? Please answer yes/no to each of the above.
   - Yes/No/Don't know

New Screen

Your responses to the following section will be visible to the group

M. Have they ever, or are they currently, looking for help to stop or reduce their cocaine use?

N. If yes, what best describes what they do or have done? [click all that apply]

a) They asked me for help or advice
b) They asked other friends or family to help them
c) They looked online for information and support
d) They consulted a medical doctor
e) They consulted another professional (e.g. counselor)
f) They looked for help from a social worker or a charity
g) They looked for advice from people who have had similar experiences to them
h) Other [please specify]

O. If known, what caused them to look for help?

P. If known, how frequently have they looked for help to reduce or stop their cocaine use?

Q. If your friend or family member that you support has ever decided to stop or reduce their cocaine use, what did you do to support them, if anything?

   i. What type of support did you offer?

   ii. Did they accept your support?

R. Are there any other types of support you would like to offer that you think could help them with their cocaine use?

S. If they didn't look for help, what was the main reason(s) why they did not look for help, in your opinion? If you do not know, please say "I don't know".

T. Did you ever look for additional help to try to reduce or stop their cocaine use?

U. If yes, what best describes what you did? [tick all that apply]

   a) I asked friends or family to help them
   b) I looked online for information and support
   c) I consulted a medical doctor on their behalf
   d) I consulted another professional (e.g. counselor) on their behalf
   e) I looked for help from a social worker or a charity
   f) I looked for advice from people who have had similar experiences to them
   g) I looked for advice from people like me who are supporting or have supported people with problems with substance misuse
   h) Other [please specify]

V. Do you think it is likely they will ever reduce or stop their cocaine use?

New Screen

A. <If patient has a formal diagnosis: check screening information> You mentioned they were diagnosed with <cocaine/substance> use disorder. Could you describe the journey to their diagnosis, if you know of anything?

If known, we are particularly interested in the following information:

- How long it took them to receive a diagnosis
- How many and what types of doctors or social workers they saw
- If they were referred to other specialists, how look the referral process took
- How they felt once they received a diagnosis
- How you felt once they received a diagnosis

So, starting off, what type of healthcare professional diagnosed them with Cocaine Use Disorder or Substance Use Disorder? Please do not tell us any information that could help identify the healthcare professional

- Please explain more

Thank you! Please click 'Next' to continue.

Thank you. That's it for this exercise. When you click 'submit' your responses will be placed on the group discussion board. Then go to 'Discussion Board for Day 4', now available on your study dashboard. Please feel free to come back at any time to see what other members of the group have written and engage in further discussion.

Day 5 Task

Welcome back and thank you for completing Day 4

Reminder: please do not mention any details that would incriminate you or anyone else, including any names or other specific details that might identify someone.

Today we are going to focus on the different treatments/therapies available for people using cocaine, some of which your friend or family member may have tried.

J. How involved are you in researching or looking for treatments/therapies for cocaine use?
    i. Would you like to be more involved?
    - Please explain more

K. Where have you looked for information about different treatments/therapies for cocaine use?
    i. Was this information easy to find?
    - How satisfied are you with these information sources?
    - Please explain more

L. How have you discussed the different treatments/therapies available for cocaine use with your friend or family member?
    i. Who starts the conversation about treatments or therapies?
    ii. How much do they allow you to be involved?
    - Please explain more

M. Which of the following treatments/therapies, if any, have they tried for their cocaine use?

	A) Please tick the treatments you have come across or researched	B) Please tick the treatments they have tried
1. Counseling/psychological support	☐	☐
2. Residential rehabilitation	☐	☐
3. Cognitive-Behavioral Therapy (CBT)	☐	☐
4. 12 Step programs (such as Narcotics anonymous)	☐	☐
5. Peer support groups	☐	☐
6. Individual and/or family therapy	☐	☐
7. Alternative medicine e.g. hypnosis, homeopathy	☐	☐
8. Medications for substance use disorder (e.g. Methadone)	☐	☐
9. Medical devices (such as electric nerve stimulators)	☐	☐
10. Lifestyle changes (complete abstinence, avoiding triggers, meditation, exercise)	☐	☐

11. Mobile apps	☐	☐
12. None of the above	☐	

N. Thinking about each treatment or therapy, we'd like you to describe:

For 8 medication, add a note: Please do not mention any specific drug names

i. If you heard of it, where did you hear about it?

ii. Did you suggest or recommend to your friend or family member you support to try this treatment or therapy? If so, why?

iii. If they tried it, why did they try it?

iv. What was their experience?

v. If they considered it but did not try it, why was this?

vi. The different pros and cons of the treatment or therapy

vii. The barriers or obstacles to the treatment or therapy

viii. Any concerns you or they may have had (please indicate if this was your concern or theirs)

Including but not limited to concerns around:

- Frequency/length of treatment or therapy
- Insurance/cost related to the treatment or therapy
- Stigma related to the treatment or therapy

i. Any input you may have had, including your level of satisfaction with the treatment or therapy options

O. Now considering all the treatments or therapies they have tried:

i. Which was their most preferred treatment or therapy?

- Why?

- Please explain more

i. Which was their most successful treatment or therapy?

- Why?
- Please explain more

Please feel free to comment on each other's responses

- What were the positives?
- [do not ask for any pharmaceutical therapies, medical devices or mobile app] What were the negatives?
- What role did you or other family or friends play, if any?
- How satisfied were you with the treatment or therapy options available?
- Did they try and discontinue treatment or therapy, if so, why?
- Do you have any concerns about the frequency or length of treatment or therapy?
- Do you have any concerns about insurance or cost related to this treatment or therapy?
- Do you have any concerns about stigma related to this treatment or therapy?
- Do they have any concerns about the frequency or length of treatment or therapy?
- Do they have any concerns about insurance or cost related to this treatment or therapy?
- Do they have any concerns about stigma related to this treatment or therapy?

If more than one treatment or therapy tried ask:

P. Did they stop a treatment or therapy to try a different treatment or therapy at any stage? If so, what lead to this decision?

i. If known, at what stage in their remission journey did they <start/ switch to> this treatment?

Q. If they received treatment or therapy, did they speak to you about their treatment or therapy experience?

Thank you! Please click 'Next' to continue.

Thank you. That's it for this exercise. When you click 'submit' your responses will be placed on the group discussion board.

Then go to 'Discussion Board for Day 5', now available on your study dashboard. Please feel free to come back at any time to see what other members of the group have written and engage in further discussion.

Day 6 Task

Welcome back and thank you for completing Day 5

Today we are going to look at your levels of satisfaction on current treatment or therapy options and how you think this could be improved.

J. On a scale of 1-10 how satisfied would you say you are with the current treatment or therapy options for cocaine use disorder
    - Why do you say that?

K. If you had to design an ideal treatment or therapy for cocaine use disorder, what would it look like?

L. From the perspective of the friend or family member that you support, what characteristics or qualities would this 'ideal therapy' have?

   Please sort into 'most important', 'somewhat important' and 'least important'
   a. Affordable (cheap and/ or potentially covered by insurance)
   b. Accessible (easy for you to get onto the therapy program e.g. easy referral)
   c. Quick (to see positive results from the therapy)
   d. Convenient (easy for you to get to)
   e. Non-stigmatizing (no negative social associations with the therapy)
   f. Helps them to manage withdrawal symptoms
   g. Helps them to manage 'cravings'
   h. High success rates (in helping patients stop their cocaine use)

   i. Safe
   j. Other [please add]

M. How do you think your friend or family member that you support would feel if a potential new treatment was taken as a pill by mouth?
   - Please explain your answer

N. If a future treatment could only improve one symptom, which symptom would you choose and why?
   - Please explain more
   - What do you mean?

O. Do you think your friend or family member that you support would choose the same or a different symptom?
   - Why do you think that?
   - Which symptom do you think they would choose?
   - Please explain more

Thank you! Please click 'Next' to continue.

Thank you. That's it for this exercise. When you click 'submit' your responses will be placed on the group discussion board.

Then go to 'Discussion Board for Day 6', now available on your study dashboard. Please feel free to come back at any time to see what other members of the group have written and engage in further discussion.

Day 7 Task

Please consider the following scenario:

“This is Alex, he uses cocaine regularly but would like to stop. He decides to look for help from a doctor who he trusts. The doctor suggests a treatment to help him control his cocaine use and Alex agrees. Alex is monitored regularly, and the doctors want to understand if the new treatment is working.”

A. Question to the group: How do you think the doctor will know if the new treatment is working?

Please feel free to comment on each other’s answers.

B. Please complete the blanks

   a) The doctor should ask Alex _______________
   b) The doctor should look out for ________________
   c) The doctor should test for _________________
   d) Alex should tell the doctor ___________________
   e) Alex should not forget to mention ________________

C. How will Alex know if the treatment is working?

D. How will Alex’s loved ones know if the treatment is working?

E. Is there anything else to consider when evaluating if the treatment is working or not?

Thank you! Please click ‘Next’ to continue.

Thank you. That’s it for this exercise. When you click ‘submit’ your responses will be placed on the group discussion board.

Then go to ‘Discussion Board for Day 7’, now available on your study dashboard. Please feel free to come back at any time to see what other members of the group have written and engage in further discussion.

Day 8 Task

Welcome back and thank you for completing Day 7!

Reminder: please do not mention any details that would incriminate you or anyone else, including any names or other specific details that might identify someone.

Today we are going to talk about clinical trials. Clinical trials are research studies performed by people that are evaluating a medical, surgical, or behavioral intervention. They help doctors understand how to treat a condition.

A. If there was a clinical trial to study an experimental treatment to help people control their cocaine use, would you consider suggesting participation to your friend or family member that you support? [note this is a hypothetical question only]

B. Please complete the blanks:
   a. A benefit of a clinical trial examining cocaine use disorder would be ___________
   b. A clinical trial for cocaine use disorder would benefit others with this disorder because ___________________
   c. A clinical trial for cocaine use disorder would benefit friends and family of those with this disorder because ____________

C. What factors do you think would stop individuals from participating in clinical trials for cocaine use disorder?

D. A clinical trial may require individuals with cocaine use disorder to take the hypothetical treatment on camera. What are your thoughts on this?

   - Do you have any concerns?
   - Do you think your friend or family member that you support would have any concerns?
   - Please explain more
   - What do you mean?

E. What do you think would make individuals feel comfortable when being monitored for the clinical trial?

(Monitoring is conducted during clinical trials to make sure participants are comfortable and safe. It may take the form of checking participant vitals, such as heart rate)

- Please explain more
- What do you mean?

F. Do you think your friend or family member that you support would be willing to participate in a future hypothetical trial that would focus on cocaine use disorder in order to test a new medication in a large-scale study?

This is a hypothetical question and will **not** result in their name being used to take part in any trials.

- Yes, I think they would be interested in participating
- Maybe, I think they would need more information
- No, I don't think they would be interested

G. How long has your friend or family member been using cocaine for?
    a. Have they ever considered stopping their cocaine use completely or considered getting treatment'/ therapy?
    b. If so:
        i. After how long since their initial cocaine use did they start to consider stopping their cocaine use?
        ii. How long was it from this initial decision to stop using cocaine to them then starting a treatment/ therapy?

H. Thank you for your participation, we understand <vendor to insert summary of findings so far>. Please could you confirm we are correct or clarify anything we have misunderstood or missed?

Thank you. That's it for this exercise. When you click 'submit' your responses will be placed on the group discussion board.

Then go to ‘Discussion Board for Day 8’, now available on your study dashboard. Please feel free to come back at any time to see what other members of the group have written and engage in further discussion.

That brings us to the end of our time together. Thank you very much for participating in the discussion!

Your answers have provided invaluable feedback and we appreciate the time you have taken. If you have any questions, please don’t hesitate to get in touch.
